# Supplementary material for: Digital twin mathematical models suggest individualized hemorrhagic shock resuscitation strategies
Source: Commun Med (Lond). 2024 Jun 12;4:113. doi: 10.1038/s43856-024-00535-6 (PMC11169363; doi:10.1038/s43856-024-00535-6)
Supplement: Supplementary file 2 — Supplementary Information [file 43856_2024_535_MOESM2_ESM.pdf]

SUPPLEMENTAL FIGURES

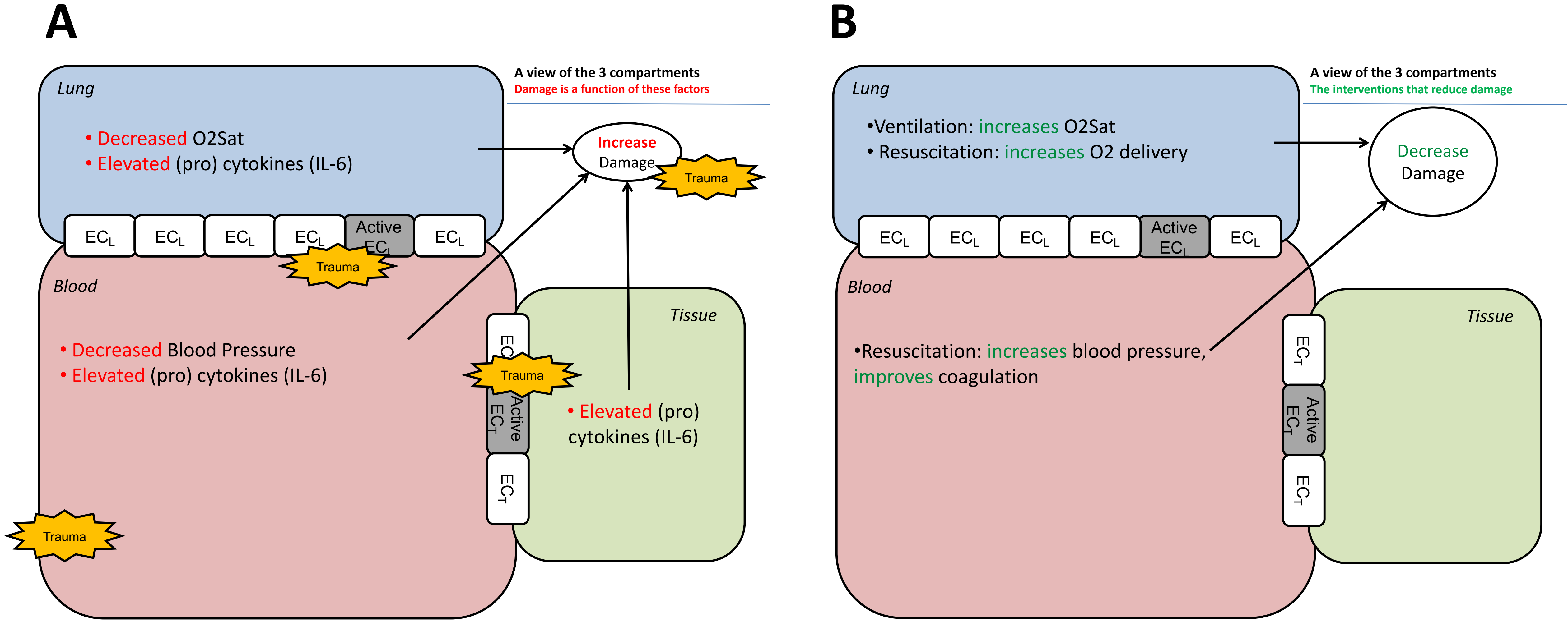

Supplementary Figure 1. Simplified model illustrating factors that influence damage. (A) Factors that lead to increased damage. Trauma initiates the activation of the four cell types (monocytes, neutrophils, lung-blood ECs, and tissue-blood ECs), and trauma additionally feeds into damage. Activated cells produce pro/anti inflammatory cytokines and precursors of NO. Pro-inflammatory cytokines feedback to promote the activation of more cells while anti-inflammatory cytokines inhibit cell activation. IL-6 feeds into damage. NO lowers blood pressure. Damage-associated molecular patterns (DAMPs) promote the activation of the tissue-blood ECs. Activated lung-blood ECs inhibit oxygen transfer from the lung to the blood, resulting in low oxygenation (O_2Sat), which also feeds into damage. (B) Factors that reduce damage. Infusions reduce damage by increasing the blood volume and thereby blood pressure including: plasma (FDP/FFP) which additionally provide inactive pro- and anti-coagulation factors; crystalloids, some of which is lost to the interstitium; red blood cells (RBCs), which enhance the process of platelets forming clots, and may play a role in oxygenation status (O_2Sat). Ventilation improves lung function and reduces damage from low O_2Sat.

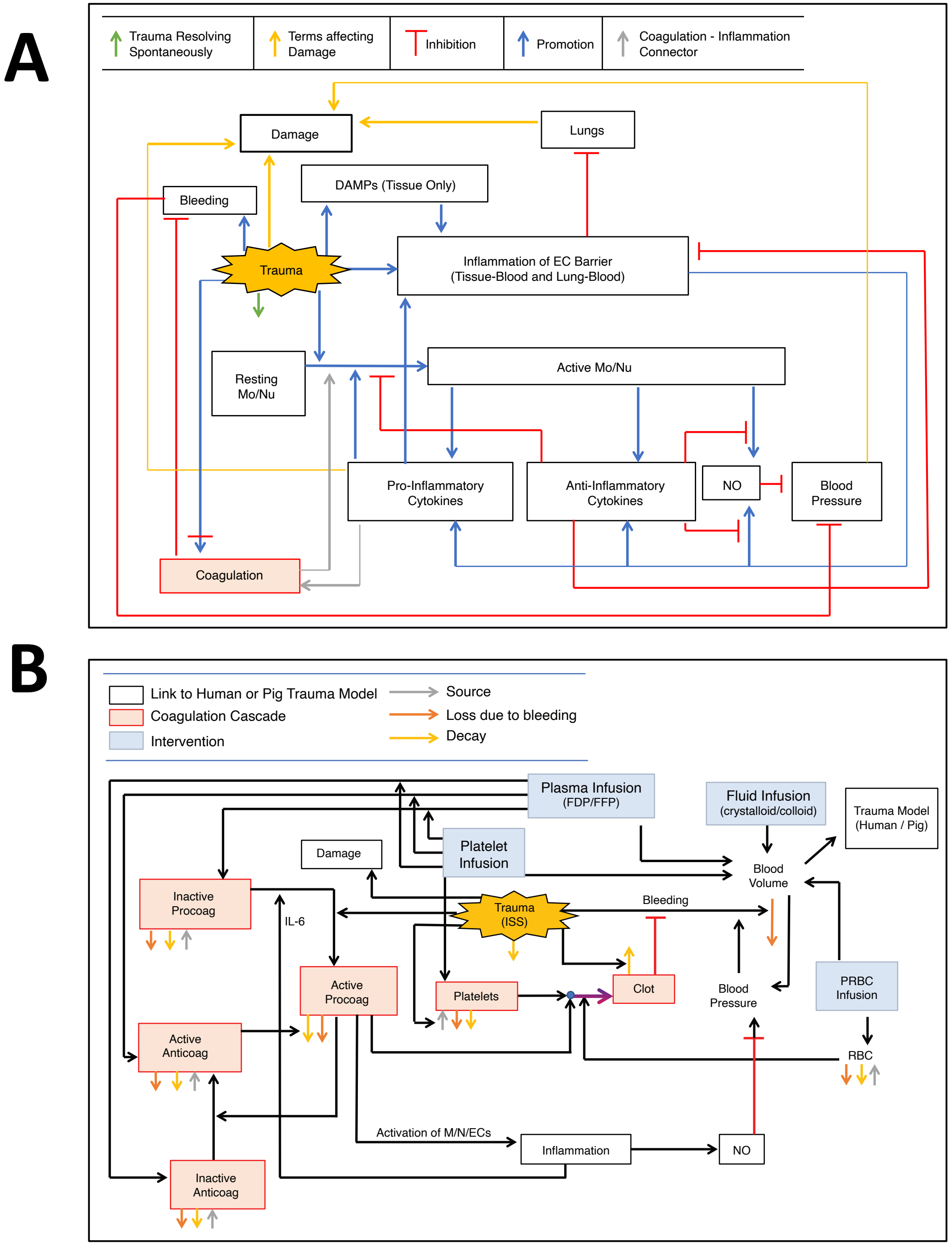

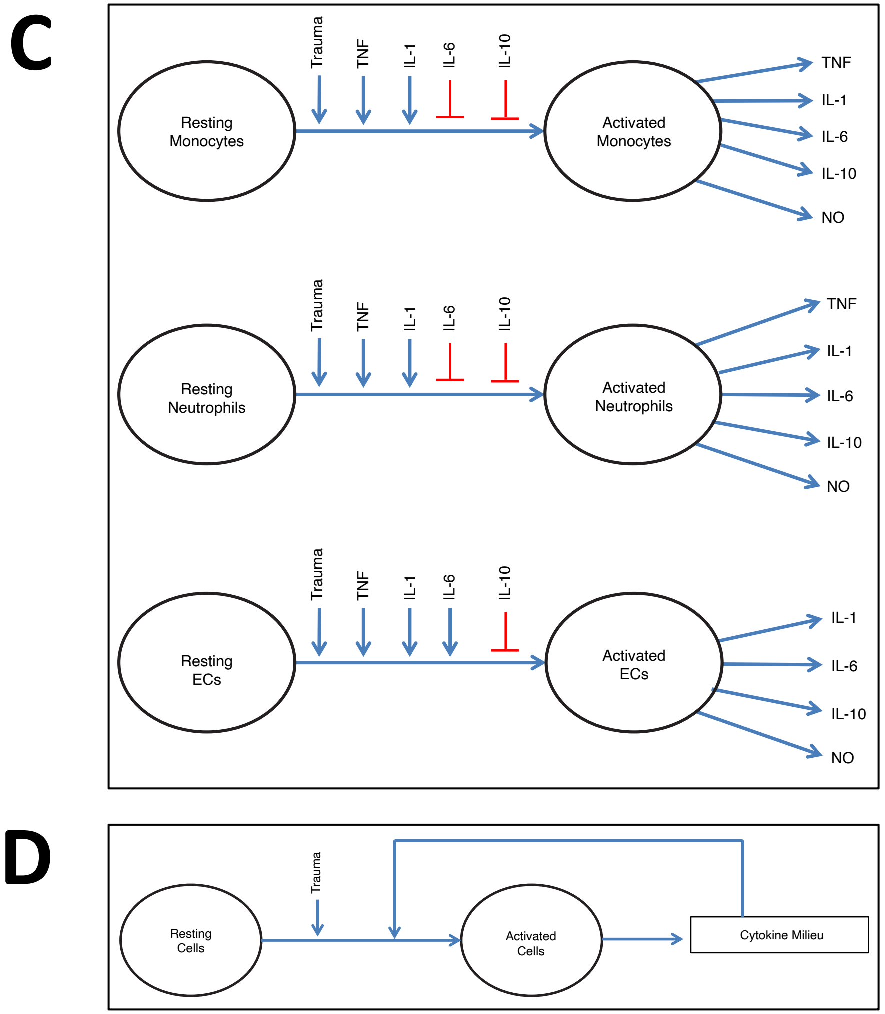

Supplementary Figure 2. Influence Diagrams. (A) Interaction among trauma, inflammation, and coagulation in the model shown in Figure 1. Damage is increased by trauma, decreased blood pressure, and inflammation; damage is decreased by interventions such as resuscitation that reverse these effects. (B) Expansion of the blood volume, blood pressure, and coagulation components in Figure. 1. (C) Crosstalk between inflammation and coagulation. (D) Resting cell activation.

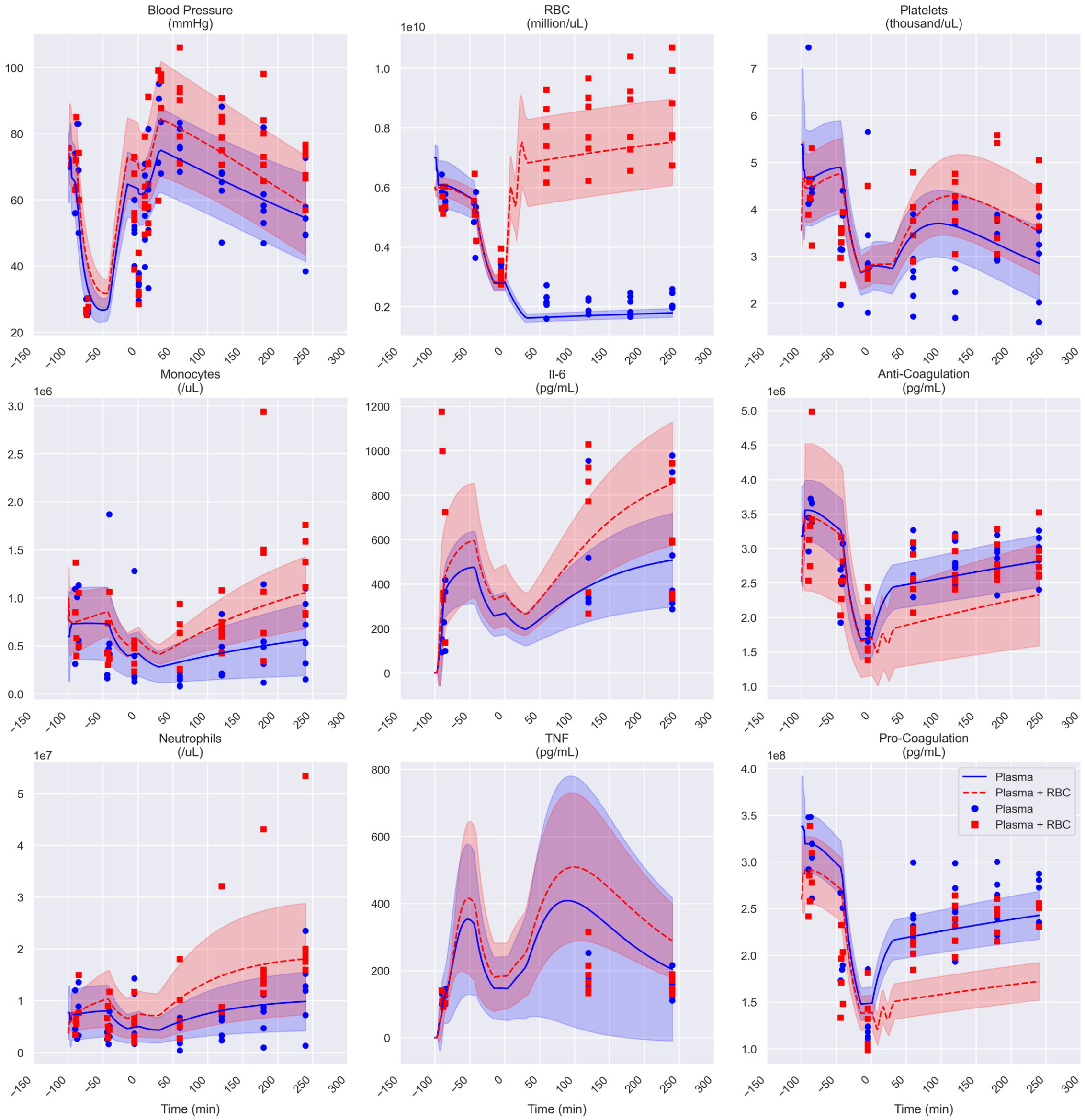

Supplementary Figure 3. Model training using animal subjects undergoing alternative resuscitation strategies: n=6 animals resuscitated with plasma only and n=6 animals resuscitated with plasma and packed red blood cells. Lines represent model fitted means and shaded areas represents the standard error of the fitted mean. Data points shown for experimental values.

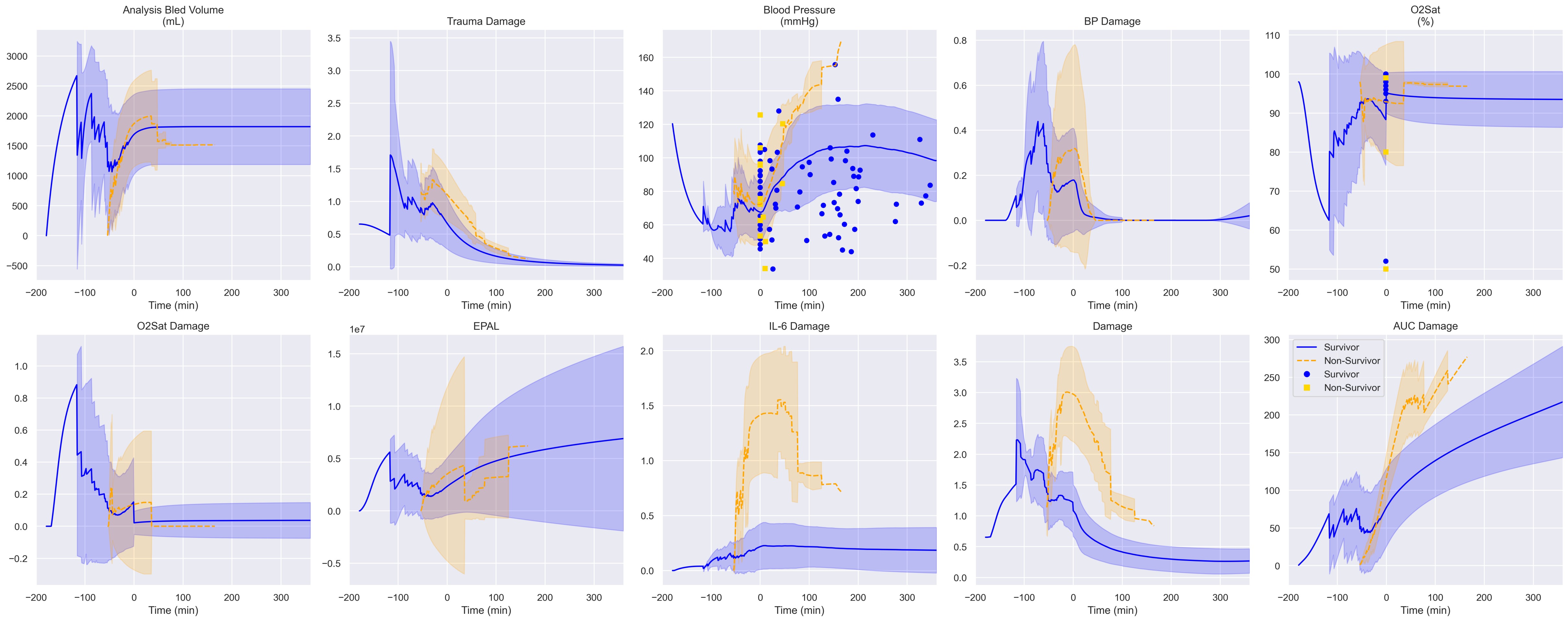

Supplementary Figure 4. Model training in n=35 patients from the PROMMTT study[27] including n=26 survivors and n=9 non-survivors. A clear distinction is seen between these groups in the "Damage" parameter as a reflection of survival vs non-survival as well as the cumulative burden of injury as reflected in "AUC Damage." Physiologic data from PROMMTT subjects is shown for Blood Pressure and *O2*Sat. All other measures are derived. Lines represent means and shaded areas represents the standard error of the mean.

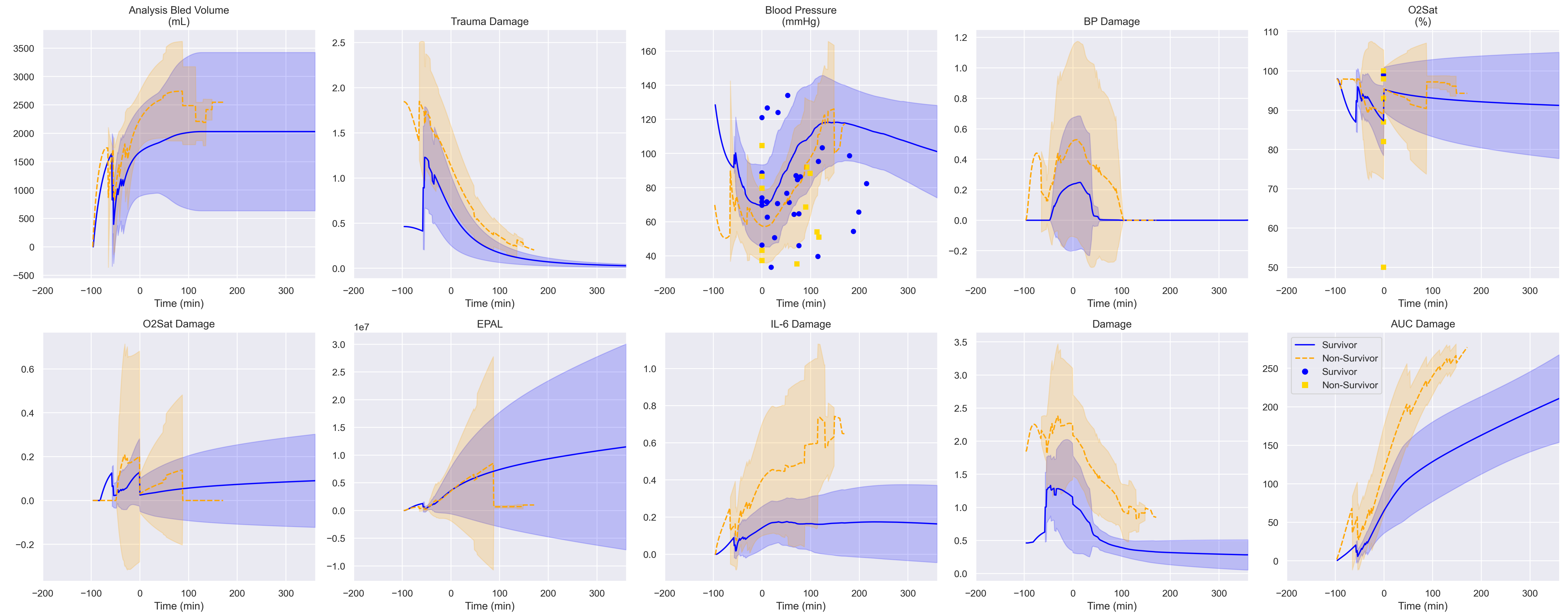

Supplementary Figure 5. Model verification in n=17 patients from the PROMMTT study[27] including n=9 survivors and n=8 non-survivors. In this model verification, a distinction is demonstrated in the "Damage" parameter as a reflection of survival vs non-survival and in the "AUC Damage" parameter as a reflection of the cumulative burden of injury. Physiologic data from PROMMTT subjects is shown for Blood Pressure and *O2*Sat. All other measures are derived. Lines represent means and shaded areas represents the standard error of the mean.

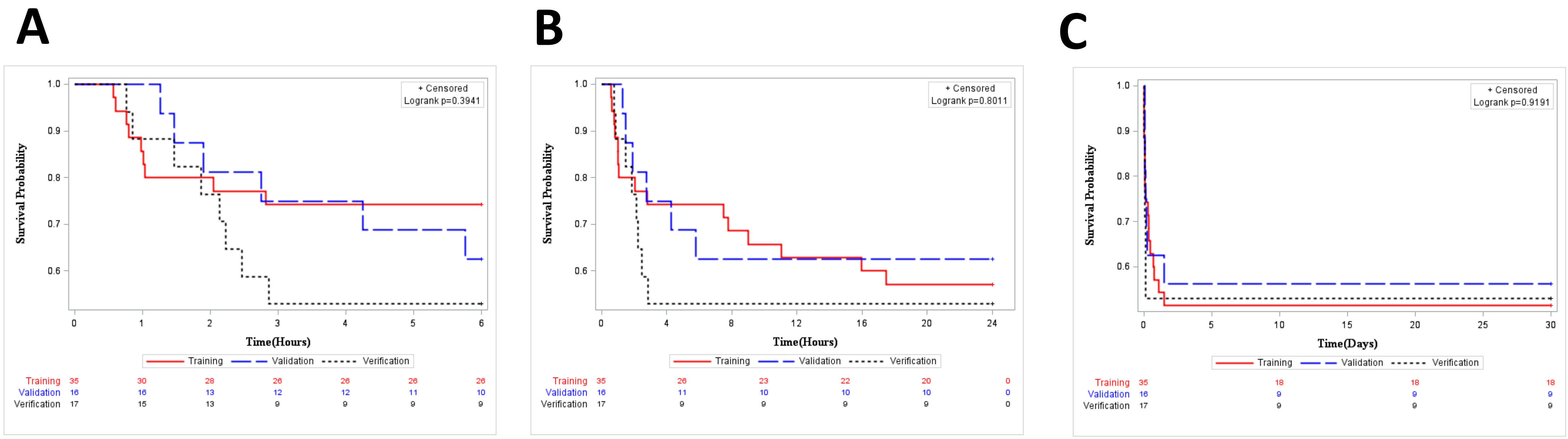

Supplementary Figure 6. Unadjusted Kaplan Meier survival analysis of patients in the PROMMTT study[27] over (A) 6-hours, (B) 24-hours, (C) 30 days. Verification patients trended towards lower early survival, but this difference was not statistically significant and did not persist over time.

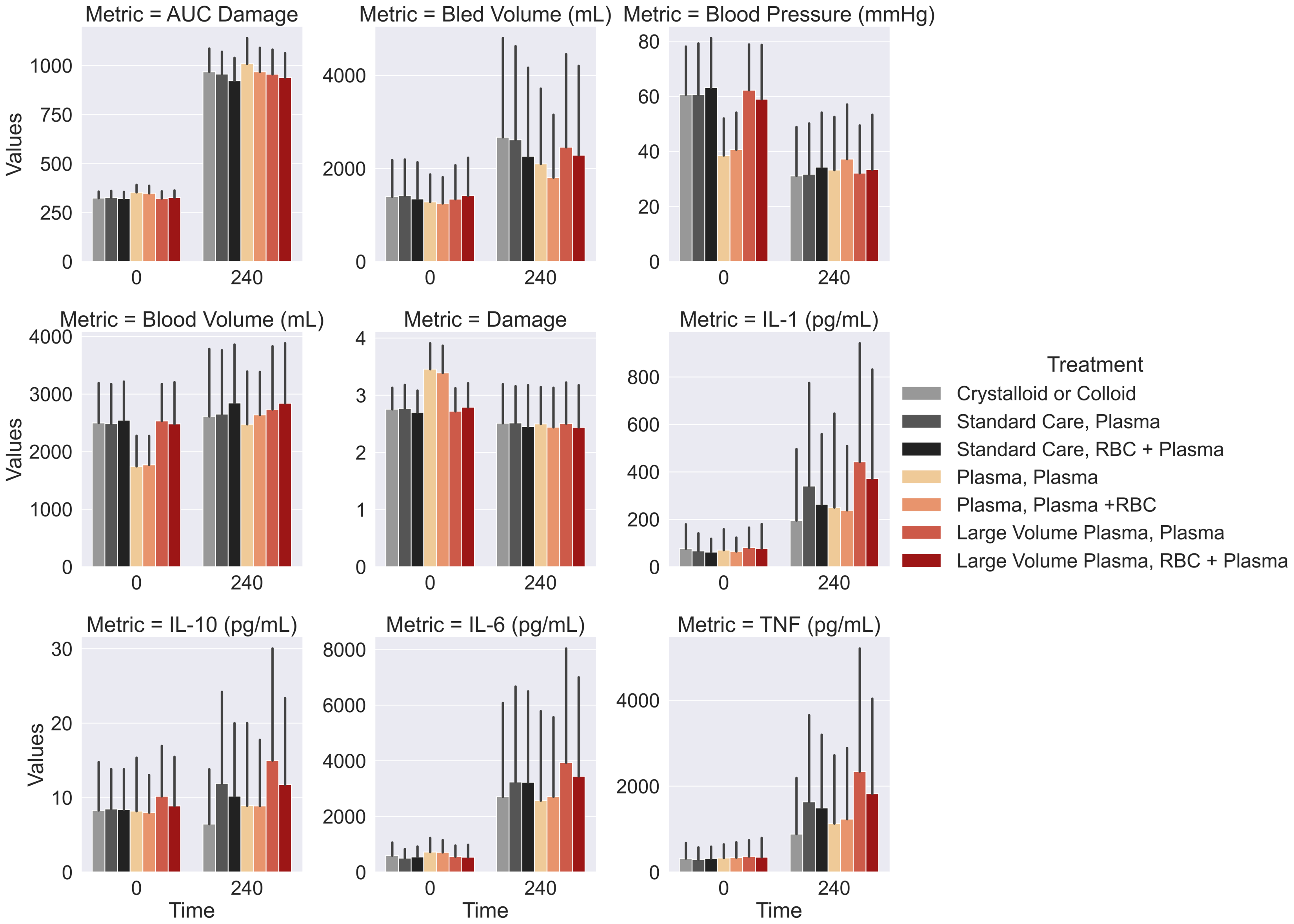

Supplementary Figure 7. Trauma simulations for 7 virtual animal populations (100 animals in each group). Low-volume pre-hospital plasma-based resuscitation led to higher levels of damage on hospital arrival and higher levels of AUC Damage at 4-hrs. This seemed to be driven by under-resuscitation pre-hospital as reflected by the low blood pressure on arrival. Conversely, increasing pre-hospital plasma volume resulted in higher levels of IL-1, IL-6, and IL-10 at 4-hrs if plasma-only resuscitation was used after arrival. Error bars represent standard error of the mean in the positive direction only for clarity. RBC, red blood cells.

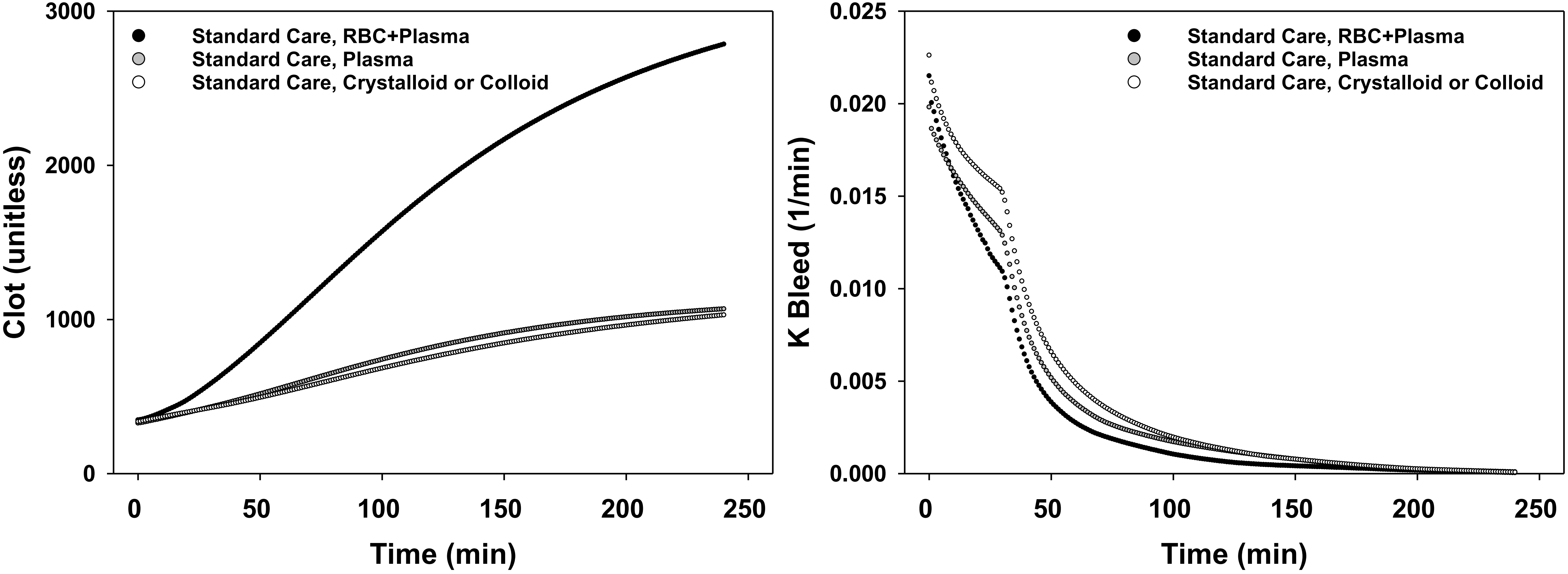

Supplementary Figure 8. Trauma simulations for 3 of 7 virtual animal populations (100 animals in each group) showed the addition of RBC+Plasma was predicted to result in increased clotting and reduced bleeding relative to the addition of plasma only and compared to the addition of crystalloid or colloid. RBC, red blood cells.

Supplemental Table 1. Characteristics of PROMMTT study subjects.

	Overall n=68	Training n=35	Verification n=17	Validation n=16	*p**
Age	35.00 [23.00, 55.00]	45.00 [25.50, 63.00]	47.00 [32.00, 55.00]	28.50 [20.75, 48.00]	0.237
Male	48 (70.6)	22 (62.9)	13 (76.5)	13 (81.2)	0.338
Blunt injury	44 (64.7)	24 (68.6)	11 (64.7)	9 (56.2)	0.694
Penetrating Injury	24 (35.3)	11 (31.4)	6 (35.3)	7 (43.8)	0.694
Injury Severity Score	26.00 [17.00, 35.00]	26.00 [20.00, 34.00]	34.00 [18.00, 41.00]	25.50 [12.25, 33.25]	0.285
ICU Days	1.00 [0.00, 5.24]	1.00 [0.00, 6.00]	0.00 [0.00, 3.00]	1.50 [0.00, 5.62]	0.687
Ventilator Days	0.00 [0.00, 2.00]	0.05 [0.00, 1.25]	0.00 [0.00, 3.00]	0.00 [0.00, 2.50]	0.835
Died in 6 hours	23 (33.8)	9 (25.7)	8 (47.1)	6 (37.5)	0.293
Died in 24 hours	29 (42.6)	15 (42.9)	8 (47.1)	6 (37.5)	0.857
Died in 30 days	32 (47.1)	17 (48.6)	8 (47.1)	7 (43.8)	0.95
Cause of Death: Exsanguination	16 (23.5)	6 (17.1)	6 (35.3)	4 (25.0)	0.346
Cause of Death: TBI	16 (23.5)	11 (31.4)	2 (11.8)	3 (18.8)	0.256
Cause of Death: Airway/Respiratory	1 (1.5)	0 (0.0)	1 (5.9)	0 (0.0)	0.218
Cause of Death: Sepsis	0 (0.0)	0 (0.0)	0 (0.0)	0 (0.0)	NA
Cause of Death: Multiorgan Failure	0 (0.0)	0 (0.0)	0 (0.0)	0 (0.0)	NA
Cause of Death: Cardiovascular	10 (14.7)	6 (17.1)	3 (17.6)	1 (6.2)	0.55
Cause of Death: Other Cause	2 (2.9)	2 (5.7)	0 (0.0)	0 (0.0)	0.379
6-hour Packed Red Blood Cells (Units)	4.00 [2.00, 5.00]	4.00 [2.00, 5.00]	4.00 [2.00, 6.00]	4.12 [2.00, 5.00]	0.872
6-hour FFP (Units)	0.00 [0.00, 1.50]	0.00 [0.00, 0.00]	0.00 [0.00, 6.00]	0.00 [0.00, 1.50]	0.495
6-hour PLT (Units)	2.00 [0.00, 4.00]	3.00 [0.00, 4.50]	1.00 [0.00, 4.00]	2.00 [0.00, 4.00]	0.631
6-hour Blood Products Total (Units)	7.00 [2.00, 12.25]	7.00 [2.00, 12.00]	5.00 [2.00, 14.00]	8.12 [3.75, 12.00]	0.985
6-hour Crystalloid (mL)	1000.00 [0.00, 2775.00]	1000.00 [0.00, 2300.00]	0.00 [0.00, 3000.00]	1000.00 [0.00, 1425.00]	0.707

FFP, fresh frozen plasma; ICU, intensive care unit; PLT, platelets; TBI, traumatic brain injury
All values shown as median [interquartile range, IQR] or as n(%)
*Kruskal-Wallis one-way ANOVA

SUPPLEMENTAL FILES

Supplementary Note 1

High Level Overview of the Model

- **The model has three compartments: blood, tissue, and lung**
- **There is an initial trauma, quantified by Injury Severity Score (ISS)**
- **Trauma initiates the activation of the four cell types** (monocytes, neutrophils, lung-blood endothelial cells [ECs], and tissue-blood EC).
- **Trauma additionally feeds into damage and also contributes to bleeding, fibrinolysis, and coagulation activation**.
- **On the trauma module side**:
  - Activated cells produce pro/anti-inflammatory cytokines and precursors of NO
    - Pro-inflammatory cytokines feedback to promote the activation of more cells. Anti-inflammatory cytokines inhibit these activation processes. IL-6 feeds into damage.
    - NO lowers blood pressure
    - Damage-associated Molecular Patterns (DAMPs, a byproduct of the initial trauma) promote activation of the tissue-blood ECs. Trauma reflected in ISS defines initial DAMP load
  - The activated lung-blood ECs inhibit oxygen transfer from the lung to the blood, resulting in low PaO2, which feeds into damage
- **On the coagulation module side**:
  - Trauma causes bleeding
    - Bleeding results in a loss of coagulation cascade components
    - Bleeding lowers blood volume, which lowers blood pressure
      - Low blood pressure reduces the rate of bleeding, while elevated blood pressure, which can occur due to infusions, can increase bleeding
      - Low blood pressure also contributes to damage
  - Trauma initiates the coagulation cascade, which is self-regulating, and leads to the formation of active coagulation factors, which combine with platelets to form clots. Additionally, trauma enhances the degradation rate of the clots, as a mechanism to represent fibrinolysis
  - Coagulation (Procoag), promotes the activation of Mo, Nu, and ECs, representing the link between coagulation and inflammation. Likewise, IL-6 (in blood) feeds back to activate additional Procoag, representing feedback from inflammation to coagulation

- **The model also supports interventions:**
  - Infusions, all of which increase the blood volume (and thereby blood pressure):
    - FDP/FFP, which additionally provide inactive pro- and anti-coagulation factors as well as active anti-coagulation factors
    - Platelet infusions provide platelets and some coagulation factors
    - Crystalloids, some of which is lost to the interstitium
    - Colloids which do not leak in to the interstitum
    - RBCs, which enhance the process of platelets forming clots, and may play a role in oxygenation status (PaO2)
  - Ventilation, which improves lung function, results in reduced damage from low-PaO2
- **The main clinical output of the model is** “**damage**”. Damage is a function of trauma, IL-6 from all the blood compartment, low PaO2, and low blood pressure.
  - Maximum damage, and time until damage is resolved are also calculated, to provide further insight into the patients’ status
- There are other model outputs as well:
  - Death: If patients experience enough cumulative damage (AUC_damage > threshold), they die
    - AUC_damage reflects the total cumulative damage
  - Survival time (last time alive)
  - Bleeding rate and bled volume

Supplementary Note 2

$$\frac{d(\text{analysis\_Bled\_Volume})}{dt} = \text{k\_bleed} \cdot \text{blood\_volume}$$

$$\begin{aligned}\frac{d(\text{blood\_volume})}{dt} = {} & \text{sum\_plasma\_infusions} + \text{sum\_RBC\_infusions} + \text{sum\_platelet\_infusions} + \text{sum\_fluid\_infusions} - \text{k\_bleed} \cdot \text{blood\_volume} \\ & + (\text{s\_blood\_volume} - \text{blood\_volume}) \cdot \text{k\_bv\_control}\end{aligned}$$

$$\begin{aligned}\frac{d(\text{blood\_pressure\_unadjusted})}{dt} = {} & (\text{s\_blood\_pressure} - \text{blood\_pressure}) \cdot \text{k\_baseline\_BloodPressure} + (\text{s\_NO} - \text{NO}) \cdot \text{blood\_pressure} \cdot \text{k\_BloodPressure\_NO} \\ & + \text{pressor\_scaler} \cdot \text{normalized\_pressor\_strength}\end{aligned}$$

$$\frac{d(\text{RBC})}{dt} = (\text{s\_RBC} - \text{RBC}) \cdot \text{k\_baseline\_RBC} + \frac{1}{\text{blood\_volume}} \cdot (\text{rbc\_infusion\_bonus\_conc} \cdot \text{s\_RBC} - \text{RBC}) \cdot \text{sum\_RBC\_infusions} - \text{k\_bleed} \cdot \text{RBC} - \frac{\text{RBC} \cdot \text{d\_BV\_dt}}{\text{blood\_volume}}$$

$$\begin{aligned}\frac{d(\text{inactive\_coag\_factor})}{dt} = {} & \text{k\_baseline\_InactiveCoagFactor} \cdot (\text{s\_inactive\_coag\_factor} - \text{inactive\_coag\_factor}) + \frac{1}{\text{blood\_volume}} \cdot (\text{plasma\_infusion\_coag\_bonus} \cdot \text{s\_inactive\_coag\_factor} \\ & - \text{inactive\_coag\_factor}) \cdot (\text{sum\_plasma\_infusions} + \text{sum\_platelet\_infusions}) - \text{k\_bleed} \cdot \text{inactive\_coag\_factor} - \text{inactive\_coag\_factor} \cdot \text{blood\_volume} \\ & \cdot (\text{k\_CoagFactorActivation\_trauma} \cdot \text{trauma} + \text{k\_CoagFactorActivation\_IL6} \cdot \text{IL6}) - \text{Inactive\_coag\_factor} \cdot \text{d\_bv\_dt} \cdot \frac{1}{\text{blood\_volume}}\end{aligned}$$

$$\begin{aligned}\frac{d(\text{active\_coag\_factor})}{dt} = {} & \text{inactive\_coag\_factor} \cdot \text{blood\_volume} \cdot (\text{k\_CoagFactorActivation\_trauma} \cdot \text{trauma} + \text{k\_CoagFactorActivation\_IL6} \cdot \text{IL6}) - \text{k\_bleed} \cdot \text{active\_coag\_factor} \\ & - \text{d\_ActiveCoagFactor} \cdot \text{active\_coag\_factor} \cdot (1 + \text{k\_ActiveCoagFactor\_ActiveAntiCoag\_enhance} \cdot \text{k\_ActiveCoagFactor\_ActiveAntiCoag\_collide} \\ & \cdot \text{active\_anti\_coag} \cdot \text{blood\_volume}) - \text{k\_InactiveAntiCoag\_ActiveCoagFactor} \cdot \text{blood\_volume} \cdot \text{inactive\_anti\_coag} \cdot \text{active\_coag\_factor} \\ & - \text{activecoagfactor\_portion\_of\_clot\_converter} \cdot \text{k\_clot} \cdot \text{blood\_volume} \cdot \text{platelets} \cdot \text{active\_coag\_factor} - \text{active\_coag\_factor} \cdot \frac{\text{d\_bv\_dt}}{\text{blood\_volume}}\end{aligned}$$

$$\begin{aligned}\frac{d(\text{inactive\_anti\_coag})}{dt} = {} & \text{k\_baseline\_InactiveAntiCoag} \cdot (\text{s\_inactive\_anti\_coag} - \text{inactive\_anti\_coag}) + \frac{1}{\text{blood\_volume}} \cdot (\text{plasma\_infusion\_coag\_bonus} \cdot \text{s\_inactive\_anti\_coag} \\ & - \text{inactive\_anti\_coag}) \cdot (\text{sum\_plasma\_infusions} + \text{sum\_platelet\_infusions}) - \text{k\_bleed} \cdot \text{inactive\_anti\_coag} - \text{k\_InactiveAntiCoag\_ActiveCoagFactor} \cdot \text{blood\_volume} \\ & \cdot \text{inactive\_anti\_coag} \cdot \text{active\_coag\_factor} - \frac{\text{inactive\_anti\_coag} \cdot (\text{d\_bv\_dt})}{\text{blood\_volume}}\end{aligned}$$

$$\begin{aligned}\frac{d(\text{active\_anti\_coag})}{dt} = {} & \text{k\_baseline\_ActiveAntiCoag} \cdot (\text{s\_active\_anti\_coag} - \text{active\_anti\_coag}) + \text{k\_InactiveAntiCoag\_ActiveCoagFactor} \cdot \text{blood\_volume} \cdot \text{inactive\_anti\_coag} \\ & \cdot \text{active\_coag\_factor} + \frac{1}{\text{blood\_volume}} \cdot (\text{plasma\_infusion\_coag\_bonus} \cdot \text{s\_active\_anti\_coag} - \text{active\_anti\_coag}) \cdot (\text{sum\_plasma\_infusions} + \text{sum\_platelet\_infusions}) \\ & - \text{k\_bleed} \cdot \text{active\_anti\_coag} - \text{k\_ActiveCoagFactor\_ActiveAntiCoag\_collide} \cdot \text{active\_coag\_factor} \cdot \text{active\_anti\_coag} \cdot \text{blood\_volume} - \frac{\text{active\_anti\_coag} \cdot \text{d\_bv\_dt}}{\text{blood\_volume}}\end{aligned}$$

$$\begin{aligned}\frac{d(\text{platelets})}{dt} = {} & \text{k\_baseline\_platelets} \cdot (\text{s\_platelets} \cdot (1 + \text{platelet\_source\_enhance}) - \text{platelets}) + \frac{1}{\text{blood\_volume}} \cdot (\text{platelet\_infusion\_bonus\_conc} \cdot \text{s\_platelets} - \text{platelets}) \\ & \cdot \text{sum\_platelet\_infusions} - \text{platelets\_portion\_of\_clot\_converter} \cdot \text{k\_clot} \cdot \text{blood\_volume} \cdot \text{platelets} \cdot \text{active\_coag\_factor} - \text{k\_bleed} \cdot \text{platelets} - \frac{\text{platelets} \cdot \text{d\_bv\_dt}}{\text{blood\_volume}}\end{aligned}$$

$$\frac{d(\text{clot})}{dt} = \text{k\_clot} \cdot \text{blood\_volume} \cdot \text{platelets} \cdot \text{active\_coag\_factor} \cdot (1 + \text{k\_clot\_RBC} \cdot \text{RBC}) - \text{d\_clot} \cdot \text{clot} \cdot (1 + \text{k\_fibrinolysis} \cdot \text{trauma})$$

$$\frac{d(\text{pe})}{dt} = -\text{d\_pe} \cdot \text{pe} + \frac{\text{load}}{\text{del}} \cdot \text{H}(\text{t} - \text{ts}) - \text{H}(\text{t} - (\text{ts} + \text{del})) + \frac{\text{load2}}{\text{del2}} \cdot \text{H}(\text{t} - \text{ts2}) - \text{H}(\text{t} - (\text{ts2} + \text{del2})) - \text{kape} \cdot \text{H}(\text{t} - \text{tapeon}) - \text{H}(\text{t} - \text{tapeoff}) \cdot \text{pe}$$

$$\frac{d(M_r)}{dt} = (\mathrm{s\_mr} \cdot (1 + \mathrm{mo\_source\_enhance}) - \mathrm{M\_r}) \cdot \mathrm{d\_Mo\_blood} - \mathrm{vma} - \mathrm{k\_bleed} \cdot \mathrm{M\_r} - \frac{\mathrm{M\_r} \cdot \mathrm{d\_bv\_dt}}{\mathrm{blood\_volume}}$$

$$\frac{d(\mathrm{Ma})}{dt} = \mathrm{v\_ma} - \mathrm{Ma} \cdot (\mathrm{d\_Mo\_blood} + \mathrm{k\_b\_t\_ma} + \mathrm{k\_bleed}) - \frac{\mathrm{Ma} \cdot \mathrm{d\_bv\_dt}}{\mathrm{blood\_volume}}$$

$$\frac{d(\mathrm{Nr})}{dt} = (\mathrm{s\_nr} \cdot (1 + \mathrm{nu\_source\_enhance}) - \mathrm{Nr}) \cdot \mathrm{d\_Nu\_blood} - \mathrm{vna} - \mathrm{k\_bleed} \cdot \mathrm{Nr} - \frac{\mathrm{Nr} \cdot \mathrm{d\_bv\_dt}}{\mathrm{blood\_volume}}$$

$$\frac{d(\mathrm{Na})}{dt} = \mathrm{v\_na} - \mathrm{Na} \cdot (\mathrm{d\_Nu\_blood} + \mathrm{k\_b\_t\_na} + \mathrm{k\_bleed}) - \frac{\mathrm{Na} \cdot \mathrm{d\_bv\_dt}}{\mathrm{blood\_volume}}$$

$$\begin{aligned}\frac{d(\mathrm{IL1})}{dt} &= \alpha\mathrm{\_1\_production} \cdot (\mathrm{k1n} \cdot \mathrm{Na} + \mathrm{k1m} \cdot \mathrm{Ma} + \mathrm{k1EP} \cdot \frac{\mathrm{EPaL} + \mathrm{EPaS}}{\mathrm{blood\_volume}}) - \mathrm{IL1} \cdot (\mathrm{d\_1} + \mathrm{k\_bleed}) - \mathrm{IL1} \cdot \mathrm{blood\_volume} \cdot (\mathrm{kn1} \cdot \frac{\mathrm{nu\_blood\_total}^2}{\mathrm{nu\_blood\_total}^2 + \mathrm{x1n}^2} \\ &+ \mathrm{km1} \cdot \frac{\mathrm{mo\_blood\_total}^2}{\mathrm{mo\_blood\_total}^2 + \mathrm{x1m}^2} + \frac{\mathrm{kEP1}}{\mathrm{blood\_volume}} \cdot (\frac{\mathrm{EPL\_total}^2}{\mathrm{EPL\_total}^2 + \mathrm{x1EP}^2} + \frac{\mathrm{EPS\_total}^2}{\mathrm{EPS\_total}^2 + \mathrm{x1EP}^2})) - \frac{\mathrm{IL1} \cdot \mathrm{d\_bv\_dt}}{\mathrm{blood\_volume}}\end{aligned}$$

$$\begin{aligned}\frac{d(\mathrm{tnf})}{dt} &= \alpha\mathrm{\_tnf\_production} \cdot (\mathrm{k\_tnfn} \cdot \mathrm{Na} + \mathrm{k\_tnfm} \cdot \mathrm{Ma}) - \mathrm{TNF} \cdot (\mathrm{d\_tnf} + \mathrm{k\_bleed}) - (\mathrm{tnf} \cdot \mathrm{blood\_volume} \cdot (\mathrm{k\_ntnf} \cdot \frac{\mathrm{nu\_blood\_total}^2}{\mathrm{nu\_blood\_total}^2 + \mathrm{x}_{\mathrm{tnfn}}^2} \\ &+ \mathrm{k\_mtnf} \cdot \frac{\mathrm{mo\_blood\_total}^2}{\mathrm{mo\_blood\_total}^2 + \mathrm{x\_tnfm}^2} + \frac{\mathrm{k\_EPtnf}}{\mathrm{blood\_volume}} \cdot (\frac{\mathrm{EPL\_total}^2}{\mathrm{EPL\_total}^2 + \mathrm{x}_{\mathrm{tnfEP}}^2} + \frac{\mathrm{EPS\_total}^2}{\mathrm{EPS\_total}^2 + \mathrm{x}_{\mathrm{tnfEP}}^2}))) - \frac{\mathrm{tnf} \cdot \mathrm{d\_bv\_dt}}{\mathrm{blood\_volume}}\end{aligned}$$

$$\begin{aligned}\frac{d(\mathrm{IL6})}{dt} &= \alpha\mathrm{\_6\_production} \cdot (\mathrm{k6n} \cdot \mathrm{Na} + \mathrm{k6m} \cdot \mathrm{Ma} + \mathrm{k6EP} \cdot (\mathrm{EPaL} + \mathrm{EPaS})/\mathrm{blood\_volume}) - \mathrm{IL6} \cdot (\mathrm{d\_6} + \mathrm{k\_bleed}) - \mathrm{IL6} \cdot \mathrm{blood\_volume} \\ &\cdot (\mathrm{pg\_per\_min\_per\_mL\_per\_cells} \cdot (\mathrm{kn6} \cdot \mathrm{nu\_blood\_total} + \mathrm{km6} \cdot \mathrm{mo\_blood\_total}) + (\mathrm{kEP6}/\mathrm{blood\_volume}) \cdot (\frac{\mathrm{EPL\_total}^2}{\mathrm{EPL\_total}^2 + \mathrm{x6EP}^2} + \frac{\mathrm{EPS\_total}^2}{\mathrm{EPS\_total}^2 + \mathrm{x6EP}^2})) \\ &- \mathrm{IL6} \cdot \frac{\mathrm{d\_bv\_dt}}{\mathrm{blood\_volume}}\end{aligned}$$

$$\begin{aligned}\frac{d(\mathrm{IL10})}{dt} &= \alpha\mathrm{\_10\_production} \cdot (\mathrm{k10n} \cdot \mathrm{Na} + \mathrm{k10m} \cdot \mathrm{Ma} + \mathrm{k10EP} \cdot (\mathrm{EPaL} + \mathrm{EPaS})/\mathrm{blood\_volume}) + (\mathrm{s\_10} - \mathrm{IL10}) \cdot \mathrm{k\_Baseline\_IL10} - \mathrm{k\_bleed} \cdot \mathrm{IL10} \\ &- \mathrm{IL10} \cdot \mathrm{blood\_volume} \cdot \mathrm{pg\_per\_cells\_per\_min} \cdot (\mathrm{one\_per\_mL} \cdot (\mathrm{kn10} \cdot \mathrm{nu\_blood\_total} + \mathrm{km10} \cdot \mathrm{mo\_blood\_total}) + (\mathrm{kEP10}/\mathrm{blood\_volume}) \\ &\cdot (\mathrm{EPL\_total} + \mathrm{EPS\_total})) - \mathrm{IL10} \cdot \frac{\mathrm{d\_bv\_dt}}{\mathrm{blood\_volume}}\end{aligned}$$

$$\begin{aligned}\frac{d(\mathrm{iNOS})}{dt} &= (\mathrm{kiNOSm} \cdot \mathrm{Ma} + \mathrm{kiNOSn} \cdot \mathrm{Na}) \cdot \frac{\mathrm{xinos10}^2}{\mathrm{il10}^2 + \mathrm{xinos10}^2} \cdot \frac{\mathrm{xiNOSNO}^2}{\mathrm{NO}^2 + \mathrm{xiNOSNO}^2} + \frac{\mathrm{kiNOSEP}}{\mathrm{blood\_volume}} \cdot (\mathrm{EPaL} \cdot \frac{(\mathrm{il10} + \mathrm{il10L})^2}{(\mathrm{il10} + \mathrm{il10L})^2 + \mathrm{xinos10}^2} \cdot \frac{\mathrm{xiNOSNO}^2}{\mathrm{NO}^2 + \mathrm{xiNOSNO}^2} \\ &+ \mathrm{EPaS} \cdot \frac{(\mathrm{il10} + \mathrm{il10S})^2}{(\mathrm{il10} + \mathrm{il10S})^2 + \mathrm{xinos10}^2} \cdot \frac{\mathrm{xiNOSNO}^2}{\mathrm{NO}^2 + \mathrm{xiNOSNO}^2}) - \mathrm{iNOS} \cdot (\mathrm{d\_iNOS} + \mathrm{k\_bleed}) - \mathrm{iNOS} \cdot \frac{\mathrm{d\_bv\_dt}}{\mathrm{blood\_volume}}\end{aligned}$$

$$\frac{d(\mathrm{eNOS})}{dt} = \frac{\mathrm{source\_eNOS} \cdot \mathrm{xeNOSpe}^2}{\mathrm{blood\_volume} \cdot (\mathrm{pe}^2 + \mathrm{xeNOSpe}^2)} - \mathrm{eNOS} \cdot (\mathrm{d\_eNOS} + \mathrm{k\_bleed}) - \mathrm{eNOS} \cdot \frac{\mathrm{d\_bv\_dt}}{\mathrm{blood\_volume}}$$

$$\frac{d(\mathrm{NO})}{dt} = \mathrm{k\_NO\_iNOS} \cdot \mathrm{iNOS} + \mathrm{k\_NO\_eNOS} \cdot \mathrm{eNOS} + \mathrm{k\_NO\_Ma} \cdot \mathrm{Ma} + \mathrm{k\_NO\_Na} \cdot \mathrm{Na} + \frac{\mathrm{k\_NO\_EP} \cdot (\mathrm{EPaL} + \mathrm{EPaS})}{\mathrm{blood\_volume}} - \mathrm{NO} \cdot (\mathrm{d\_NO} + \mathrm{k\_bleed} + \frac{\mathrm{d\_bv\_dt}}{\mathrm{blood\_volume}})$$

$$\frac{d(\mathrm{mrL})}{dt} = (\mathrm{s\_mr} \cdot (1 + \mathrm{mo\_source\_enhance\_L}) - \mathrm{mrL}) \cdot \mathrm{d\_Mr\_tis\_lung} - \mathrm{vmaL}$$

$$\frac{d(\mathrm{MaL})}{dt} = \mathrm{vmaL} - \mathrm{MaL} \cdot \mathrm{d\_Ma\_tis\_lung} + \mathrm{k\_b\_t\_ma} \cdot (\mathrm{k\_b\_t\_ma\_L}) \cdot \left(\frac{\mathrm{ma} \cdot \mathrm{blood\_volume}}{\mathrm{v\_Lecf}}\right)$$

$$\frac{d(\mathrm{nrL})}{dt} = (\mathrm{s\_nr} \cdot (1 + \mathrm{nu\_source\_enhance\_L}) - \mathrm{nrL}) \cdot \mathrm{d\_Nu\_tis\_lung} - \mathrm{vnaL}$$

$$\frac{d(\mathrm{naL})}{dt} = \mathrm{vnaL} - \mathrm{naL} \cdot \mathrm{d\_Nu\_tis\_lung} + \mathrm{k\_b\_t\_na} \cdot (\mathrm{k\_b\_t\_na\_L}) \cdot (\mathrm{na} \cdot \mathrm{blood\_volume}/\mathrm{v\_Lecf})$$

$$\frac{d(\mathrm{EPrL})}{dt} = -\mathrm{vEPaL} - \mathrm{d\_EP} \cdot (\mathrm{EPrL} - \mathrm{s\_EPrL})$$

$$\frac{d(\mathrm{EPaL})}{dt} = \mathrm{vEPaL} - \mathrm{d\_EP} \cdot \mathrm{EPaL}$$

$$\frac{d(\mathrm{il1L})}{dt} = \alpha\mathrm{\_1\_production}\left(\mathrm{k1n} \cdot \mathrm{naL} + \mathrm{k1m} \cdot \mathrm{maL} + \frac{\mathrm{k1EP} \cdot \mathrm{EPaL}}{\mathrm{v\_Lecf}}\right) - \mathrm{d\_1} \cdot \mathrm{il1L} - \left(\mathrm{IL1L} \cdot \mathrm{v\_Lecf}\left(\mathrm{kn1} \cdot \frac{\mathrm{nu\_lungtotal}^2}{\mathrm{nu\_lungtotal}^2 + \mathrm{x1n}^2} + \mathrm{km1} \cdot \frac{\mathrm{mo\_lungtotal}^2}{\mathrm{mo\_lungtotal}^2 + \mathrm{x1m}^2}\right.\right.$$
$$\left.\left. + \frac{\mathrm{kEP1}}{\mathrm{v\_Lecf}} \cdot \frac{\mathrm{EPL\_total}^2}{\mathrm{EPL\_total}^2 + \mathrm{x1EP}^2}\right)\right)$$

$$\frac{d(\mathrm{tnfL})}{dt} = \alpha\mathrm{\_tnf\_production}\,(\mathrm{k\_tnfn} \cdot \mathrm{naL} + \mathrm{k\_tnfm} \cdot \mathrm{maL}) - \mathrm{d\_tnf} \cdot \mathrm{tnfL} - \left(\mathrm{tnfL} \cdot \mathrm{v\_Lecf}\left(\mathrm{k\_ntnf} \cdot \frac{\mathrm{nu\_lung\_total}^2}{\mathrm{nu\_lung\_total}^2 + \mathrm{x\_tnfn}^2} + \mathrm{k\_mtnf} \cdot \frac{\mathrm{mo\_lung\_total}^2}{\mathrm{mo\_lung\_total}^2 + \mathrm{x\_tnfm}^2}\right.\right.$$
$$\left.\left. + \frac{\mathrm{k\_EPtnf}}{\mathrm{v\_Lecf}} \cdot \frac{\mathrm{EPL\_total}^2}{\mathrm{EPL\_total}^2 + \mathrm{x\_tnfEP}^2}\right)\right)$$

$$\frac{d(\mathrm{il6L})}{dt} = \alpha\mathrm{\_6\_production}\left(\mathrm{k\_6n} \cdot \mathrm{naL} + \mathrm{k\_6m} \cdot \mathrm{maL} + \frac{\mathrm{k\_6EP} \cdot \mathrm{EPaL}}{\mathrm{v\_Lecf}}\right) - \mathrm{d\_6} \cdot \mathrm{il6L} - \left(\mathrm{IL6L} \cdot \mathrm{v\_Lecf}\left(\mathrm{pg\_per\_min\_per\_mL\_per\_cells}\left(\mathrm{k\_n6} \cdot \mathrm{nu\_lung\_total}\right.\right.\right.$$
$$\left.\left.\left. + \mathrm{k\_m6} \cdot \mathrm{mo\_lung\_total}\right) + \frac{\mathrm{k\_EP6}}{\mathrm{v\_Lecf}} \cdot \frac{\mathrm{EPL\_total}^2}{\mathrm{EPL\_total}^2 + \mathrm{x\_6EP}^2}\right)\right)$$

$$\frac{d(\mathrm{IL10L})}{dt} = \alpha\mathrm{\_10\_production} \cdot \left(\mathrm{k\_10n} \cdot \mathrm{naL} + \mathrm{k\_10m} \cdot \mathrm{maL} + \frac{\mathrm{k\_10EP} \cdot \mathrm{EPaL}}{\mathrm{v\_Lecf}}\right) + \mathrm{k\_baseline\_IL10} \cdot (\mathrm{s\_10} - \mathrm{IL10L}) - \mathrm{IL10L} \cdot \mathrm{v\_Lecf}$$
$$\cdot \mathrm{pg\_per\_cells\_per\_min} \cdot \left(\mathrm{one\_per\_mL} \cdot (\mathrm{k\_n10} \cdot \mathrm{nu\_lung\_total} + \mathrm{k\_m10} \cdot \mathrm{mo\_lung\_total}) + \frac{\mathrm{k\_EP10}}{\mathrm{v\_Lecf}} \cdot \mathrm{EPL\_total}\right)$$

$$\frac{d(\mathrm{damp})}{dt} = -\mathrm{damp} \cdot \mathrm{k\_EPdamp} \cdot \frac{\mathrm{EPS\_total}^2}{\mathrm{EPS\_total}^2 + \mathrm{xdampEP}^2}$$

$$\frac{d(\mathrm{mrS})}{dt} = (\mathrm{s\_mr} \cdot (1 + \mathrm{mo\_source\_enhance\_s}) - \mathrm{mrS}) \cdot \mathrm{d\_Mr\_tis\_lung} - \mathrm{vmaS}$$

$$\frac{d(\mathrm{MaS})}{dt} = \mathrm{v\_maS} - \mathrm{MaS} \cdot \mathrm{d\_Ma\_tis\_lung} + \mathrm{k\_b\_t\_ma} \cdot (1 - \mathrm{k\_b\_t\_ma\_L}) \cdot (\mathrm{ma} \cdot \mathrm{blood\_volume}/\mathrm{v\_Secf})$$

$$\frac{d(\mathrm{nrS})}{dt} = \mathrm{s\_nr}\,(1 + \mathrm{nu\_source\_enhance\_S}) - \mathrm{nrS} \cdot \mathrm{d\_Nu\_tis\_lung} - \mathrm{v\_naS}$$

$$\frac{d(\mathrm{naS})}{dt} = \mathrm{v\_naS} - \mathrm{naS} \cdot \mathrm{d\_Nu\_tis\_lung} + \mathrm{k\_b\_t\_na}\,(1 - \mathrm{k\_b\_t\_na\_L}) \cdot \frac{\mathrm{na} \cdot \mathrm{blood\_volume}}{\mathrm{v\_Secf}}$$

$$\frac{d(\mathrm{EPrS})}{dt} = -\mathrm{v\_EPaS} - \mathrm{d\_EP} \cdot (\mathrm{EPrS} - \mathrm{s\_EPrS})$$

$$\frac{d(\mathrm{EPaS})}{dt} = \mathrm{v\_EPaS} - \mathrm{d\_EP} \cdot \mathrm{EPaS}$$

$$\frac{d(\mathrm{il1S})}{dt} = \alpha\mathrm{\_1\_production}\left(\mathrm{k\_1n} \cdot \mathrm{naS} + \mathrm{k\_1m} \cdot \mathrm{maS} + \frac{\mathrm{k\_1EP} \cdot \mathrm{EPaS}}{\mathrm{v\_Secf}}\right) - \mathrm{d\_1} \cdot \mathrm{il1S} - \mathrm{IL1S} \cdot \mathrm{v\_Secf}\left(\mathrm{kn1} \cdot \frac{\mathrm{nu\_splanchnic\_total}^2}{\mathrm{nu\_splanchnic\_total}^2 + \mathrm{x1n}^2}\right.$$
$$\left. + \mathrm{km1} \cdot \frac{\mathrm{mo\_splanchnic\_total}^2}{\mathrm{mo\_splanchnic\_total}^2 + \mathrm{x1m}^2} + \frac{\mathrm{k\_EP1}}{\mathrm{v\_Secf}} \cdot \frac{\mathrm{EPS\_total}^2}{\mathrm{EPS\_total}^2 + \mathrm{x1EP}^2}\right)$$

$$\frac{d(tnfS)}{dt} = \alpha\_tnf\_production\,(k\_tnfn \cdot naS + k\_tnfm \cdot maS) - d\_tnf \cdot tnfS - tnfS \cdot v\_Secf\left(k\_ntnf \cdot \frac{nu\_splanchnic\_total^2}{nu\_splanchnic\_total^2 + x\_tnfn^2}\right.$$

$$\left. + k\_mtnf \cdot \frac{mo\_splanchnic\_total^2}{mo\_splanchnic\_total^2 + x\_tnfxm^2} + \frac{k\_EPtnf}{v\_Secf} \cdot \frac{EPS\_total^2}{EPS\_total^2 + x\_tnfEP^2}\right)$$

$$\frac{d(il6S)}{dt} = \alpha\_6\_production\left(k\_6n \cdot naS + k\_6m \cdot maS + \frac{k\_6EP \cdot EPaS}{v\_Secf}\right) - d\_6 \cdot il6S - IL6S \cdot v\_Secf\,(pg\_per\_min\_per\_mL\_per\_cells\,(k\_n6 \cdot nu\_splanchnic\_total$$

$$+ k\_m6 \cdot mo\_splanchnic\_total) + \frac{k\_EP6}{v\_Secf} \cdot \frac{EPS\_total^2}{EPS\_total^2 + x\_6EP^2}\Bigg)$$

$$\frac{d(il10S)}{dt} = \alpha\_10\_production\left(k\_10n \cdot naS + k\_10m \cdot maS + \frac{k\_10EP \cdot EPaS}{v\_Secf}\right) + k\_baseline\_IL10\,(s\_10 - IL10S)$$

$$- IL10S \cdot v\_Secf \cdot pg\_per\_cells\_per\_min\left(one\_per\_mL\,(k\_n10 \cdot nu\_splanchnic\_total + k\_m10 \cdot mo\_splanchnic\_total) + \frac{k\_EP10}{v\_Secf} \cdot EPS\_total\right)$$

$$\frac{d(AUC\_Damage)}{dt} = Damage$$

Supplementary Note 3

ODE Dictionary

Letter	Usage	Example
d	decay	d_ActiveCoagFactor = decay of active coagulation fact[illegible]
k	rate	k_bleed = rate of bleeding
s	initial steady state	s_blood pressure = initial blood pressure
t	time	ton_plasma_infusion1 = time the first plasma infusion
v	inhibitory Hill function parameter	
x	pro Hill function parameter	

Supplementary Note 4

Parameters

Section: _infusions_manual

Parameter	Pig Value	Human Value	Unit	Notes
min_plasma_dose	100	100		
ton_plasma_infusion1	0	0	min	
t_plasma_infusion_duration1	0	0	min	
plasma_dose1	0	0	mL	
ton_plasma_infusion2	0	0	min	
t_plasma_infusion_duration2	0	0	min	
plasma_dose2	0	0	mL	
ton_plasma_infusion3	0	0	min	
t_plasma_infusion_duration3	0	0	min	
plasma_dose3	0	0	mL	
ton_plasma_infusion4	0	0	min	
t_plasma_infusion_duration4	0	0	min	
plasma_dose4	0	0	mL	
ton_plasma_infusion5	0	0	min	
t_plasma_infusion_duration5	0	0	min	
plasma_dose5	0	0	mL	
ton_plasma_infusion6	0	0	min	
t_plasma_infusion_duration6	0	0	min	
plasma_dose6	0	0	mL	
ton_plasma_infusion7	0	0	min	
t_plasma_infusion_duration7	0	0	min	
plasma_dose7	0	0	mL	
ton_plasma_infusion8	0	0	min	
t_plasma_infusion_duration8	0	0	min	
plasma_dose8	0	0	mL	
ton_plasma_infusion9	0	0	min	
t_plasma_infusion_duration9	0	0	min	
plasma_dose9	0	0	mL	
ton_plasma_infusion10	0	0	min	
t_plasma_infusion_duration10	0	0	min	
plasma_dose10	0	0	mL	
plasma_infusion_coag_bonus	1.55E+00	1.55E+00	1	Plasma infusion bags have 55% higher conc than healthy blood.
min_fluid_dose	100	100		
ton_fluid_infusion1	0	0	min	
t_fluid_infusion_duration1	0	0	min	
fluid_dose1	0	0	mL	
ton_fluid_infusion2	0	0	min	
t_fluid_infusion_duration2	0	0	min	
fluid_dose2	0	0	mL	
ton_fluid_infusion3	0	0	min	
t_fluid_infusion_duration3	0	0	min	
fluid_dose3	0	0	mL	
ton_fluid_infusion4	0	0	min	
t_fluid_infusion_duration4	0	0	min	
fluid_dose4	0	0	mL	
ton_fluid_infusion5	0	0	min	
t_fluid_infusion_duration5	0	0	min	
fluid_dose5	0	0	mL	
ton_fluid_infusion6	0	0	min	
t_fluid_infusion_duration6	0	0	min	
fluid_dose6	0	0	mL	
ton_fluid_infusion7	0	0	min	
t_fluid_infusion_duration7	0	0	min	
fluid_dose7	0	0	mL	
ton_fluid_infusion8	0	0	min	
t_fluid_infusion_duration8	0	0	min	
fluid_dose8	0	0	mL	
ton_fluid_infusion9	0	0	min	
t_fluid_infusion_duration9	0	0	min	
fluid_dose9	0	0	mL	
ton_fluid_infusion10	0	0	min	
t_fluid_infusion_duration10	0	0	min	
fluid_dose10	0	0	mL	
min_RBC_dose	100	100		
ton_RBC_infusion1	0	0	min	
t_RBC_infusion_duration1	0	0	min	
RBC_dose1	0	0	mL	
ton_RBC_infusion2	0	0	min	
t_RBC_infusion_duration2	0	0	min	
RBC_dose2	0	0	mL	
ton_RBC_infusion3	0	0	min	
t_RBC_infusion_duration3	0	0	min	

RBC_dose3	0	0	mL	
ton_RBC_infusion4	0	0	min	
t_RBC_infusion_duration4	0	0	min	
RBC_dose4	0	0	mL	
ton_RBC_infusion5	0	0	min	
t_RBC_infusion_duration5	0	0	min	
RBC_dose5	0	0	mL	
ton_RBC_infusion6	0	0	min	
t_RBC_infusion_duration6	0	0	min	
RBC_dose6	0	0	mL	
ton_RBC_infusion7	0	0	min	
t_RBC_infusion_duration7	0	0	min	
RBC_dose7	0	0	mL	
ton_RBC_infusion8	0	0	min	
t_RBC_infusion_duration8	0	0	min	
RBC_dose8	0	0	mL	
ton_RBC_infusion9	0	0	min	
t_RBC_infusion_duration9	0	0	min	
RBC_dose9	0	0	mL	
ton_RBC_infusion10	0	0	min	
t_RBC_infusion_duration10	0	0	min	
RBC_dose10	0	0	mL	
rbc_infusion_bonus_conc	1.44E+00	1.44E+00		pRBC bags have 44% higher conc than healthy blood.
min_platelet_dose	100	100		
ton_platelet_infusion1	0	0	min	
t_platelet_infusion_duration1	0	0	min	
platelet_dose1	0	0	mL	
ton_platelet_infusion2	0	0	min	
t_platelet_infusion_duration2	0	0	min	
platelet_dose2	0	0	mL	
ton_platelet_infusion3	0	0	min	
t_platelet_infusion_duration3	0	0	min	
platelet_dose3	0	0	mL	
ton_platelet_infusion4	0	0	min	
t_platelet_infusion_duration4	0	0	min	
platelet_dose4	0	0	mL	
ton_platelet_infusion5	0	0	min	
t_platelet_infusion_duration5	0	0	min	
platelet_dose5	0	0	mL	
ton_platelet_infusion6	0	0	min	
t_platelet_infusion_duration6	0	0	min	
platelet_dose6	0	0	mL	
ton_platelet_infusion7	0	0	min	
t_platelet_infusion_duration7	0	0	min	
platelet_dose7	0	0	mL	
ton_platelet_infusion8	0	0	min	
t_platelet_infusion_duration8	0	0	min	
platelet_dose8	0	0	mL	
ton_platelet_infusion9	0	0	min	
t_platelet_infusion_duration9	0	0	min	
platelet_dose9	0	0	mL	
ton_platelet_infusion10	0	0	min	
t_platelet_infusion_duration10	0	0	min	
platelet_dose10	0	0	mL	
platelet_infusion_bonus_conc	3.67E+00	3.67E+00		Platelet bags have 367% conc compared to healthy blood.

Section: _infusions_optimizable

Parameter	Pig Value	Human Value	Unit	Notes
plasma_dose_total	0	0		
fluid_dose_total	0	0		
RBC_dose_total	0	0		
Platelet_dose_total	0	0		

Section: _blood pressure

Parameter	Pig Value	Human Value	Unit	Notes
k_baseline_BloodPressure	1.00E-03	1.86E-03		
k_BloodPressure_NO	1.00E-07	3.59E-07	L/(umol*min)	
v_Lecf	5.00E+02	5.00E+02	mL	
v_Secf	5.00E+03	5.00E+02	mL	

Section: _Blood Volume and Pressure

Parameter	Pig Value	Human Value	Unit	Notes
pressor_scaler	-	1.00E+01	mmHg/min	Used a patient+fit_scenario to make it slightly stronger than NO

Section: _Coagulation Species

Parameter	Pig Value	Human Value	Unit	Notes
ISS	1	5.00E+01	1	Injury Severity Score
s_blood_volume	5.00E+03	5.00E+03	mL	
s_blood_pressure	6.50E+01	6.50E+01	mmHg	
s_RBC	5.00E+09	5.00E+09	RBC/mL	
s_inactive_coag_factor	1.00E+08	1.00E+08	pg/mL	
s_inactive_anti_coag	4.00E+06	4.00E+06	pg/mL	
s_active_anti_coag	1.00E+03	1.00E+03	pg/mL	The starting natural pool should be smaller than that of the inactive form.
s_platelets	3.00E+00	3.00E+00	platelets/mL	Starting pool should be on the scale of 150,000,000-450,000,000, but we are taking platelets to be multiplied by 1e-8 (in data as well)

Section: _Trauma

Parameter	Pig Value	Human Value	Unit	Notes
Second_Trauma_ISS_par	15	0.00E+00		Femur fracture in pigs; No second injury in humans
Third_Trauma_ISS_par	9	0.00E+00		Liver injury in pigs; No third injury in humans

Section: _activation rates

Parameter	Pig Value	Human Value	Unit	Notes
k_CoagFactorActivation_trauma	1.00E-07	4.15E-10	1/(min*mL)	
k_InactiveAntiCoag_ActiveCoagFactor	1.00E-07	2.45E-11	1/(pg*min)	

Section: _rates of return to baseline

Parameter	Pig Value	Human Value	Unit	Notes
k_baseline_InactiveCoagFactor	2.41E-04	2.41E-04	1/min	
k_baseline_InactiveAntiCoag	1.44E-03	1.44E-03	1/min	
k_baseline_ActiveAntiCoag	2.77E-02	2.77E-02	1/min	From "Yang 2005 and Heeb 1992"
k_baseline_rbc	4.01E-06	4.01E-06	1/min	From "D'Alessandro 2010"
k_baseline_platelets	1.20E-04	1.20E-04	1/min	

Section: ._trauma, clotting, and bleeding

Parameter	Pig Value	Human Value	Unit	Notes
recovery_slowness	1.00E+04	1.00E+04	min^2	Can optionally be varied per patient; Larger values make trauma recovery slower
k_clot	1.00E-09	1.00E-09	mL/(pg*platelets*min)	
k_clot_RBC	1.00E-07	5.43E-08	mL/RBC	
k_bleed_trauma	1.20E+01	12	1	
k_bleed_control	5.00E-04	5.00E-04		
k_bv_control	1.00E-02	2.57E-03	1/min	Global fit parameter
k_fibrinolysis	1.00E-03	7.78E-06		

Section: _death and source

Parameter	Pig Value	Human Value	Unit	Notes
d_ActiveCoagFactor	6.93E-01	6.93E-01	1/min	From "Ann Thorac Surg 2006;82:2315-2322" and "Arteriosclerosis, Thrombosis, and Vascular Biology. 2005;25:2463"
d_clot	1.20E+01	6.05E-06	1/min	
k_ActiveCoagFactor_ActiveAntiCoag_enhance	0.001	1.78	min	
k_ActiveCoagFactor_ActiveAntiCoag_collide	0.001	3.27E-02	1/(pg*min)	
k_platelet_source_enhance	0.001	1.25E+03		
max_iss	75	75		Maximum Injury Severity Score

Section: _Connections to trauma model

Parameter	Pig Value	Human Value	Unit	Notes
k_CoagFactorActivation_IL6	1.00E-07	5.93E-12	1/(pg*min)	
kmCoag	1.00E-04	7.71E-07	1/(min*mL)	
xmCoag	1.00E-05	2.15E+02	pg/mL	
knCoag	1	6.92E-07	1/(min*mL)	
xnCoag	1.00E-09	4.35E+04	pg/mL	
kEPCoag	1.00E-04	9.29E-07	1/min	
xEPCoag	1.00E-05	3.84E+06	pg	

Section: Scaled Outputs

Parameter	Pig Value	Human Value	Unit	Notes
s_pig_active_coag_factor	24000	-		

Section: _MONOCYTES

Parameter	Pig Value	Human Value	Unit	Notes
k_b_t_ma0	1.00E-03	1.85E-05	1/(cells*min)	
trauma_cell_activation_scale	10	4.39E-08	1/(min*mL)	Scale this 75/20 to 20 in fits
s_mr	8.00E+04	8.00E+04	cells/mL	Calculated as average wbc
d_Mo_blood	2.41E-04	2.41E-04	1/min	Death rate of Mo in the blood, resting and active forms are assumed to have the same death rate; From "Yang 2014"

d_Mr_tis_lung	1.58E-05	1.58E-05	1/min	Death rate of RESTING Mo in the tissue and lung, unlike in blood, resting and active have different values; Fom "Principles of Surgical Practice"
d_Ma_tis_lung	6.88E-05	6.88E-05	1/min	Death rate of ACTIVE Mo in the tissue and lung, unlike in blood, resting and active have different values
k_mo_source_enhance	0.01	0.01	mL*min	

Section: _NEUTROPHILS

Parameter	Pig Value	Human Value	Unit	Notes
k_b_t_na0	0.001	1.90E-08	1/(cells*min)	
s_nr	2.50E+06	2.50E+06	cells/mL	Calculated as average wbc
d_Nu_blood	1.93E-03	1.93E-03	1/min	Death rate of Nu in the blood, resting and active forms are assumed to have the same death rate; From "Dancey 1976" and "Tak 2013"
d_Nu_tis_lung	2.41E-04	2.41E-04	1/min	Death rate of Nu in the tissue and lung, unlike in monocyte, resting and active forms are assumed to have the same death rate; From "Tak 2013"
k_nu_source_enhance	0.01	0.01	mL*min	
S_EPrL	2.02E+10	2.02E+10	cells	
S_EPrS	2.02E+10	2.02E+10	cells	popgen par -> [1e9, 1e11], softbounds(1e10, 3e10). Base value is taken from lung value above.
d_EP	7.91E-06	1.00E-03	1/min	

Section: CELL MIGRATION

Parameter	Pig Value	Human Value	Unit	Notes
k_b_t_ma_L	0.4	0.018	1	Percentage of the migrating monocytes that go to the lung, the rest go to splanchnic
k_b_t_na_L	0.4	0.701	1	Percentage of the migrating neutrophils that go to the lung, the rest go to splanchnic

Section: _Per Patient Variability Pars (for Popgen)

Parameter	Pig Value	Human Value	Unit	Notes
alpha_damp_sens	1	1	1	
alpha_tnf_production	1	1	1	
alpha_1_production	1	1	1	
alpha_6_production	1	1	1	
alpha_10_production	1	1	1	

Section: _DAMPS

Parameter	Pig Value	Human Value	Unit	Notes
damp_load	0.001	7.79E-04	pg	
kEPdamp	1	8.00E-06	1/min	
xEPdamp	7.80E+04	1.67E+03	pg	
xdampEP	100	2.60E+10		

Section: _TNF

Parameter	Pig Value	Human Value	Unit	Notes
ktnfm	1.00E+07	4.81E-02	pg/(cells*min)	
ktnfn	1.00E+07	2.77E-03		
d_tnf	4.08E-02	4.08E-02	1/min	From "Giannoudis 2004"
kmtnf	10	6.20E-07	1/(min*mL)	
kntnf	1.00E-04	6.83E-07		
kEPtnf	1.00E-07	1.96E-07		
xmtnf	10	6.13E+02	pg/mL	
xntnf	10	2.86E+05		
xEPtnf	20	5.25E+05		
xtnfEP	1.00E-05	1.10E+09	cells	
xtnfn	1	9.30E+05		
xtnfm	1	1.15E+06		

Section: _IL-1

Parameter	Pig Value	Human Value	Unit	Notes
k1m	1.00E+05	4.74E-02	pg/(cells*min)	
k1n	1.00E+05	1.24E-03		
k1EP	1.00E+05	1.16E-06		
d_1	1.16E-01	1.16E-01	1/min	From "Giannoudis 2004"
km1	100	6.97E-07	1/(min*mL)	
kn1	1.00E-04	1.07E-06		
kEP1	1.00E-07	9.20E-07		
x1n	1	8.76E+06	cells/mL	
x1m	1	1.60E+05		

x1EP	2	5.95E+08		
xm1	0.10	4.21E+02	pg/mL	
xn1	0.10	1.06E+05		
xEP1	1.00E-05	1.63E+08		
k6m	0.01	1.65E-01	pg/(cells*min)	
k6n	0.01	4.63E-04		
k6EP	5.00E-07	6.82E-09		
d_6	2.89E-03	2.89E-03	1/min	
km6	1.00E-14	4.11E-15	mL/pg	
kn6	1.00E-14	1.02E-14		
kEP6	1.00E-10	1.35E-05		
xEP6	500	3.81E+05	pg	
xm6	1	8.35E-22	1	
xn6	1	1.20E-21		
x6EP	1	1.44E+09	cells	
s_10	10	10	pg/mL	
k10m	1.00E+03	3.98E+02	pg/(cells*min)	
k10n	1.00E+03	4.59E+01		
k10EP	1.00E+03	6.34E-08		
km10	1.00E-06	4.96E-06	mL/pg	
kn10	1.00E-06	1.11E-06		
kEP10	1.00E-06	9.60E-08		
k_Baseline_IL10	3.85E-03	3.85E-03		The IL-10 decay rate from "Fuchs 1996"
xep10	1.00E-14	1.10E-02	1	Set based on "[il10*bv + avg(il10L, il10S)*avg(lung_vol, tissue_vol)]^2", if that is 1e14, use 1e-14.
xm10	2.50E-07	6.72E-03	1	Similarly to xep10, xm10 and xn10 should be based just on "avg(il10, il10L, il10S)^2"
xn10	2.50E-07	4.82E-03	1	Similarly to xep10, xm10 and xn10 should be based just on "avg(il10, il10L, il10S)^2"
kape	0	0		
load	0	0		
load2	0	0		
d_pe	2.307	2.307	1/min	
lps_dose	0	0		
tapeoff	0	0		
tapeon	0	0		
del	1	1		
ts	0	0		
del2	0.1	0.1		
ts2	0	0		

Section: _NO AND PRECURSORS

Parameter	Pig Value	Human Value	Unit	Notes
kinosm	1	7.70E+00		
kinosn	1	1.20E+01		
kiNOSEP	1	2.49E+01		
xinos10	1	0.79	pg/mL	
xinosno	1	0.27		
xenospe	25	25		
d_enos	1.03E-05	1.03E-05	1/min	Enos and inos decay rates are fit, and use the heuristic that the inos value is greater than the enos value
d_iNOS	1.01E-05	1.01E-05	1/min	Enos and inos decay rates are fit, and use the heuristic that the inos value is greater than the enos value
d_NO	2.06E-03	2.06E-03	1/min	From "Clin Exp Pharm Physiol 2004 31:591, Himeno 2004 (5.6 hrs), or Kelm 1999 (NO: 0.05-1 s; Nitrite: 110 s; Nitrate: 5-8 hr)"
source_enos	1.00E-03	1.00E-03		
k_NO_iNOS	0.1	62.48	1/min	
k_NO_eNOS	0.1	10.73		
k_NO_Ma	0.1	3.21		
k_NO_Na	0.1	5.72E-06		
k_NO_EP	0.1	7.53E-06		

Section: VENTILATION

Parameter	Pig Value	Human Value	Unit	Notes
ton_vents	-	0		Marks the time the vents turn on; only plays a role if vent_switch

t_vents_off	0	0		Should be set in patient scenario if they were on vents
O2Sat0	98	98	1	
vent_par1	2.50E+06	2.50E+06	cells	
vent_par2	1.00E-05	50	1	
vent_par2	1.00E-04	-		

Section: StO2

Parameter	Pig Value	Human Value	Unit	Notes
k_stO2_bv	7.50E-04	7.50E-04		
k_vent	1.00E-03	1.00E-03		
k_stO2_EPaL	1.00E-07	1.00E-07		
k_stO2_EPaL_vent	5	5		
stO20	98	98		
t_vents_on	t_initial	0		Zero by default in humans, t_initial for pigs

Section: Method and Settings

Parameter	Pig Value	Human Value	Unit	Notes
t_initial	-21	-21	min	popgen par
t_final	7200	7200	min	

Section: CLINICAL OUTPUTS

Parameter	Pig Value	Human Value	Unit	Notes
k_damage_il6	1.25	1.25	1/min	popgen par
x_damage_il6	90	90		
IL6_damage_threshold	10	10		
use_blood_il6_only	1	-		Set to 1 if you want to use blood il6 only in the damage calculation
k_Damage_O2Sat	2.5	2.5		popgen par
O2Sat_Damage_threshold	93	93		
max_o2sat	98	98		While technically possible to achieve o2sat
k_damage_bp	2.5	2.5		popgen par
x_damage_bp	37	37		
bp_damage_threshold	70	70		
k_damage_trauma	3.75	3.75	1/min	popgen par
damage_death_threshold	-	276.53		Tuned and then locked via PROMMTT data

Section: _unit conversions

Parameter	Pig Value	Human Value	Unit	Notes
mL_min	0	0		Used in fluid infusions "else(0)" case
pg_per_min_per_mL_per_cells	1	1	pg/(min*mL*cells)	Used in IL6 consumption to move from activation formulation
one_per_mL	1	1	1/mL	Used in mo/nu consumption of il10
pg_per_cells_per_min	1	1	pg/(cells*min)	Used in general consumption of il10
unit_mL	1	1	mL	Used in trauma activation of EPs, to change units without changing value of rates used for Mo and Nu.
one_per_min	0	0	1/min	Used in the urination event
activecoagfactor_portion_of_clot_converter	1	1	pg/mL	Used to give active_coag_factor correct units
platelets_portion_of_clot_converter	1	1	platelets/mL	Used to give platelets correct units

Section: _unit conversions

Parameter	Pig Value	Human Value	Unit	Notes
guiscaleil10	5000	5000		
guiscaleil6	20000	20000		
guiscaletnf	18000	18000		
guiscaleil1	100	100		
GuiScaleDamage	0	0		

Supplementary Note 5

Initial Conditions

Initial Coagulation Conditions				
Parameter	Pig Value	Human Value	Unit	Notes
ISS	25	50	1	Injury Severity Score
blood_volume	5.00E+03	5.00E+03	mL	Listed as blood_volume(0) =s_blood_volume
blood_pressure	65	65	mmHg	Listed as blood_pressure(0) =s_blood_pressure
RBC	5.00E+09	5.00E+09	RBC/mL	Listed as RBC(0) =s_RBC
inactive_coag_factor	1.00E+08	1.00E+08	pg/mL	Listed as inactive_coag_factor(0) =s_inactive_coag_factor
active_coag_factor	0	0	pg/mL	
inactive_anti_coag	4.00E+06	4.00E+06	pg/mL	Listed as inactive_anti_coag(0) =s_inactive_anti_coag
active_anti_coag	1.00E+03	1.00E+03	pg/mL	Listed as active_anti_coag(0) =s_active_anti_coag
platelets	3	3	platelets/mL	Listed as platelets(0) =s_platelets
clot	0	0	1	

Initial Trauma Conditions				
Parameter	Pig Value	Human Value	Unit	Notes
mr	8.00E+03	8.00E+04	cells/mL	Listed as mr(0) = s_mr. Value from s_mr applied
ma	0	0	cells/mL	
mrL	8.00E+03	8.00E+04	cells/mL	Listed as mrL(0) = s_mr
maL	0	0	cells/mL	
mrS	8.00E+03	8.00E+04	cells/mL	Listed as mrS(0) = s_mr
maS	0	0	cells/mL	
nr	2.50E+06	2.50E+06	cells/mL	Listed as nr(0) = s_nr
na	0	0	cells/mL	
nrL	2.50E+06	2.50E+06	cells/mL	Listed as nrL(0) = s_nr
naL	0	0	cells/mL	
nrS	2.50E+06	2.50E+06	cells/mL	Listed as nrS(0) = s_nr
naS	0	0	cells/mL	
EPrL	2.02E+10	2.02E+10	cells	Listed as EPrL(0) = s_EPrL
EPaL	0	0	cells	
EPrS	2.02E+10	2.02E+10	cells	Listed as EPrS(0) = s_EPrS
EPaS	0	0	cells	
tnf	0	0	pg/mL	
tnfL	0	0	pg/mL	
tnfS	0	0	pg/mL	
il1	0	0	pg/mL	
il1L	0	0	pg/mL	
il1S	0	0	pg/mL	
il6	0	0	pg/mL	
il6L	0	0	pg/mL	
il6S	0	0	pg/mL	
il10	10	10	pg/mL	Listed as il10(0) = s_10
il10L	10	10	pg/mL	Listed as il10(0) = s_10
il10S	10	10	pg/mL	Listed as il10(0) = s_10
iNOS	0	0	umol/L	
eNOS	0.02	0.019352991	umol/L	eNOS(0) is listed as s_eNOS, which itself is assigned as source_enos/(d_eNOS*s_blood_volume), further evaluated as 0.001 / (1.03343201E-05*5000)
NO	0.969491085	100.6506298	umol/L	NO is listed as s_NO, which itself is assigned as k_NO_eNOS*s_eNOS/d_NO, further evaluated as 10.7288849*0.01935299062 /0.002062938
