# Supplementary material for: Digital twin mathematical models suggest individualized hemorrhagic shock resuscitation strategies
Source: Commun Med (Lond). 2024 Jun 12;4:113. doi: 10.1038/s43856-024-00535-6 (PMC11169363; doi:10.1038/s43856-024-00535-6)
Supplement: Supplementary file 3 — Description of Additional Supplementary Files [file 43856_2024_535_MOESM3_ESM.pdf]

Description of Additional Supplementary Files

File name- Supplementary Data 1

File description- Model Parameters
