# Supplementary material for: Digital twin mathematical models suggest individualized hemorrhagic shock resuscitation strategies
Source: Commun Med (Lond). 2024 Jun 12;4:113. doi: 10.1038/s43856-024-00535-6 (PMC11169363; doi:10.1038/s43856-024-00535-6)
Supplement: Supplementary file 5 — Reporting Summary [file 43856_2024_535_MOESM5_ESM.pdf]

nature portfolio

Corresponding author(s): Jeremy W. Cannon, MD, SM

Last updated by author(s): Jan 30, 2024

Reporting Summary

Nature Portfolio wishes to improve the reproducibility of the work that we publish. This form provides structure for consistency and transparency in reporting. For further information on Nature Portfolio policies, see our Editorial Policies and the Editorial Policy Checklist.

Statistics

For all statistical analyses, confirm that the following items are present in the figure legend, table legend, main text, or Methods section.

n/a	Confirmed	
☐	☒	The exact sample size (*n*) for each experimental group/condition, given as a discrete number and unit of measurement
☐	☒	A statement on whether measurements were taken from distinct samples or whether the same sample was measured repeatedly
☐	☒	The statistical test(s) used AND whether they are one- or two-sided<br>*Only common tests should be described solely by name; describe more complex techniques in the Methods section.*
☐	☒	A description of all covariates tested
☐	☒	A description of any assumptions or corrections, such as tests of normality and adjustment for multiple comparisons
☐	☒	A full description of the statistical parameters including central tendency (e.g. means) or other basic estimates (e.g. regression coefficient) AND variation (e.g. standard deviation) or associated estimates of uncertainty (e.g. confidence intervals)
☐	☒	For null hypothesis testing, the test statistic (e.g. F, t, r) with confidence intervals, effect sizes, degrees of freedom and P value noted<br>*Give P values as exact values whenever suitable.*
☒	☐	For Bayesian analysis, information on the choice of priors and Markov chain Monte Carlo settings
☒	☐	For hierarchical and complex designs, identification of the appropriate level for tests and full reporting of outcomes
☒	☐	Estimates of effect sizes (e.g. Cohen's *d*, Pearson's *r*), indicating how they were calculated

Our web collection on statistics for biologists contains articles on many of the points above.

Software and code

Policy information about availability of computer code

Data collection	Data used for this analysis was taken from two prior publications. Animal data was from Spoerke et al (Spoerke N, Zink K, Cho SD, Differding J, Muller P, Karahan A, Sondeen J, Holcomb JB, Schreiber M. Lyophilized plasma for resuscitation in a swine model of severe injury. Arch Surg. 2009 Sep;144(9):829-34. doi: 10.1001/archsurg.2009.154. PMID: 19797107.) and human data was from Holcomb et al. (Holcomb JB, del Junco DJ, Fox EE, Wade CE, Cohen MJ, Schreiber MA, Alarcon LH, Bai Y, Brasel KJ, Bulger EM, Cotton BA, Matijevic N, Muskat P, Myers JG, Phelan HA, White CE, Zhang J, Rahbar MH; PROMMTT Study Group. The prospective, observational, multicenter, major trauma transfusion (PROMMTT) study: comparative effectiveness of a time-varying treatment with competing risks. JAMA Surg. 2013 Feb;148(2):127-36. doi: 10.1001/2013.jamasurg.387. PMID: 23560283; PMCID: PMC3740072.)
Data analysis	All statistical analysis was performed using R version 4.0.3 (R Foundation for Statistical Computing, Vienna, Austria. https://www.R-project.org/).

For manuscripts utilizing custom algorithms or software that are central to the research but not yet described in published literature, software must be made available to editors and reviewers. We strongly encourage code deposition in a community repository (e.g. GitHub). See the Nature Portfolio guidelines for submitting code & software for further information.

Data

Policy information about availability of data

All manuscripts must include a data availability statement. This statement should provide the following information, where applicable:

- Accession codes, unique identifiers, or web links for publicly available datasets
- A description of any restrictions on data availability
- For clinical datasets or third party data, please ensure that the statement adheres to our policy

The study protocol and data (both human and animal) used for this study will be made available upon reasonable request.

Human research participants

Policy information about studies involving human research participants and Sex and Gender in Research.

Reporting on sex and gender	Animals were female juvenile Yorkshire crossbred swine. Sex of the humans was available and is provided. These study subjects were selected at random and thus the sex of the patients reported reflect the overall population of the original study. A sex-based analysis was not the primary focus of this report
Population characteristics	These are provided in Supplemental Table 1.
Recruitment	Study subject recruitment was performed by the investigators of the original PROMMTT study. All subjects with complete data suitable for modeling were included in our model.
Ethics oversight	Human Research Protection Office, U.S. Army Medical Research and Materiel Command

Note that full information on the approval of the study protocol must also be provided in the manuscript.

Field-specific reporting

Please select the one below that is the best fit for your research. If you are not sure, read the appropriate sections before making your selection.

☒ Life sciences ☐ Behavioural & social sciences ☐ Ecological, evolutionary & environmental sciences

For a reference copy of the document with all sections, see nature.com/documents/nr-reporting-summary-flat.pdf

Life sciences study design

All studies must disclose on these points even when the disclosure is negative.

Sample size	Animal study: All 16 animals with sufficiently granular data available for modeling were used in this study.<br>Human Study: A total of n=68 patients with complete data on our parameters of interest were identified from the n=905 original PROMMTT study patients. This number allowed for an adequate sample size to train the model (n=35) and then to verify the model constants (n=17) and then to validate the model (n=16).
Data exclusions	Animal study modeling: Of the 32 animals in the original study, 16 were monitored with sufficient granularity to qualify for inclusion in this modeling study. Human study modeling: Of the 905 original study patients, 68 had sufficiently granular data and biologically plausible outcomes that made them suitable for modeling. All of these patients were included.
Replication	After tuning the model, the results were verified for both animal and human studies.
Randomization	Animals and patients were randomly assigned to training, verification, and validation groups.
Blinding	All study animals survived the investigation so outcome blinding was not considered. Investigators were unblinded to the outcome of included human study subjects in order to ensure inclusion of a balanced number of survivors and non-survivors.

Reporting for specific materials, systems and methods

We require information from authors about some types of materials, experimental systems and methods used in many studies. Here, indicate whether each material, system or method listed is relevant to your study. If you are not sure if a list item applies to your research, read the appropriate section before selecting a response.

Materials & experimental systems

n/a	Involved in the study
☒	☐ Antibodies
☒	☐ Eukaryotic cell lines
☒	☐ Palaeontology and archaeology
☒	☐ Animals and other organisms
☐	☒ Clinical data
☒	☐ Dual use research of concern

Methods

n/a	Involved in the study
☒	☐ ChIP-seq
☒	☐ Flow cytometry
☒	☐ MRI-based neuroimaging

Clinical data

Policy information about clinical studies

All manuscripts should comply with the ICMJE guidelines for publication of clinical research and a completed CONSORT checklist must be included with all submissions.

Clinical trial registration	The original PROMMTT study was registered as ClinicalTrials.gov Identifier: NCT01545232 (https://clinicaltrials.gov/ct2/show/NCT01545232)
Study protocol	The study protocol for the modeling study will be made available upon reasonable request.
Data collection	All data used in this modeling study had been previously collected.
Outcomes	Primary simulated outcomes included blood loss, time of death (in non-survivors) and a composite "damage" metric composed of the following elements: trauma severity, high IL-6, low oxygen (O2) saturation, and low blood pressure.
